# An Efficient 3D-Printed Gravity Mixer for Lab-on-a-CD Applications

**DOI:** 10.3390/mi15030291

**Published:** 2024-02-20

**Authors:** Yunxia Wang, Yong Zhang, Zheng Qiao, Wanjun Wang

**Affiliations:** Department of Mechanical Engineering, Louisiana State University, Baton Rouge, LA 70803, USA; yxwang321@gmail.com (Y.W.); zyff0534@gmail.com (Y.Z.); zqiao1@lsu.edu (Z.Q.)

**Keywords:** gravity mixer, centrifugal, 3D printing, lab-on-chip, microfluidic platform

## Abstract

We introduced a new, highly efficient, and uncomplicated mixing device for centrifugal microfluidic platforms, called the gravity mixer. The gravity mixer featured a slope channel that can precisely and sequentially control micro-volume liquids using centrifugal, capillary, and gravitational forces to achieve the desired mixing effect. By adjusting the angular velocity, micro-volumes of liquids in the slope channel of the gravity mixer could be precisely controlled across a wide range. We evaluated the change in mixing efficiency by varying the slope geometry, including the slope angle and the number of mixing cycles. Our study of gravity mixers with different slope angles revealed that the 80° angle gravity mixer achieved the best mixing efficiency, with a standard deviation of 2.39. Additionally, the mixing process in the gravity mixer is highly repeatable, achieving the desired mixing efficiency after only three cycles of operation. Our gravity mixer design and implementation can facilitate the development of more complex 3D-printed lab-on-chip devices.

## 1. Introduction

Centrifugal microfluidic discs, also known as Lab-on-a-CD (LOCD), have found diverse applications in biomedical and chemical analyses, like point-of-care testing [1,2,3,4]. LOCD is a compact disk that eliminates the need for external micropumps and can be easily controlled by a rotational motor. Mounted on a rotor shaft, the CD’s angular motion produces centrifugal force, replacing the micropump and controlling various modules on the LOCD. The LOCD’s ease of use has made it a popular platform for biomedical and chemical analyses, such as cell lysis and enzymatic studies [5,6], and DNA analysis [7]. In comparison with traditional chemistry tests, LOCD devices are compact, automatically operated, enable fast and highly sensitive detection, and consume fewer reagents [8]. Consequently, LOCDs have emerged as a technology with high potential to impact the food, agriculture, and biosystems fields [9].

Currently, the most common techniques for fabricating LOCD devices include soft lithography, etching, computer numerical control (CNC) micro-milling, and laser-directed writing [10,11,12,13]. These techniques are typically utilized and manufactured in laboratories, requiring professional operators, a long processing time, and costly processes, as well as specialized infrastructure like clean rooms. However, modern additive manufacturing (AM) techniques have emerged to transfer most of the fabrication skills to 3D printers [14]. Three-dimensional printing technologies simplify the development process and reduce the required infrastructure to a single platform. Moreover, AM provides the ability to build truly three-dimensional (3D) architectures that are highly desirable for microfluidic purposes [15,16]. Consequently, AM may replace conventional fabricating techniques like soft lithography as the ideal technique of choice for rapid prototyping. Currently, the most practical and popular AM technologies are fused deposition modelling (FDM), stereolithography (SLA), poly-jet, selective laser sintering, two-photon lithography, and layered hydro-spinning [17]. SLA, as the world’s first 3D printing technology, was invented in the 1980s. It relies on the selective curing of resin using UV light [18]. Compared with other 3D printing technologies, SLA offers a fine resolution, within a range enough to reproduce the designs of complex 3D microfluidic mixers. Specifically, SLA-printed parts can be completely solid and impervious to water intrusion [19], making them better suited for underwater applications than FDM-printed parts that are subject to void formation [20]. Digital light processing (DLP) and liquid crystal display (LCD) are two other types of resin printers, where the liquid photopolymer is cured or solidified using light from a projector and an array of LEDs, respectively. DLP and LCD are like SLA, but LCD is the latest and cheapest technology compared with SLA and DLP. In our study, LCD 3D printing technology was used.

Microfluidic functions like fluidic transportation, metering, valving, and mixing are essential in LOCD devices used in various fields [21]. Microfluidic mixing is a particularly important and effective technique in LOCD for studying biochemical kinetics, synthesis, protein folding, and more [22,23,24,25,26,27,28,29,30,31,32]. Microfluidic mixers are indispensable components in LOCD devices for faster reagent homogenization and chemical or biological reactions [33]. As these fields continue to grow, there is a need for more multifunctional and multipurpose microfluidic mixers. Mixing at the microscale in LOCD devices can be classified into active and passive mixing. Active mixing always requires an external power source, such as ultrasonic waves [34], electrowetting [35], magnetic fields [36], or periodically changing flow rates [37], and a control system to enhance mixing. While active mixing can significantly enhance mixing efficiency, it requires additional design and construction of force-driving devices or structures, making it more complex and expensive. In contrast, passive mixing only depends on the geometric design of the LOCD, utilizing the hydrodynamic energy produced by a pressure difference via a pump, centrifugal or gravity force, to promote mixing performance [38]. Thus, a simple passive micromixer without any extra components, like electrodes or magnets, is more appropriate than an intricate active mixer if it can achieve a better mixing effect. Moreover, a simple, passive mixer is portable, inexpensive, and easy to use and manufacture, making it more easily integrated with LOCD devices.

The flows of microfluidic systems typically have small Reynolds numbers, meaning that most flows are laminar and turbulent, and the convective mixing of two liquids is not likely. In laminar flow, it can take around 500 s [39] for species to diffuse 1 mm in water, making rapid and thorough mixing a challenging task. To improve the mixing efficiencies, many different types of micromixers for use in LOCD have been developed, with some of the representative ones including a reciprocating flow-based centrifugal microfluidics mixer, curved centrifugal micromixer and centrifugal serpentine micromixers, etc. [40,41,42,43]. They are normally fabricated using lithography and CNC (computer numerical control) technologies. These passive mixers have some common drawbacks, including complex designs, complicated operation systems, and not ideal mixing efficiencies. It is therefore highly desirable to develop a new, low-cost passive mixer with a sufficient mixing performance, short mixing time, and simpler design.

In this paper, we presented a microfluidic passive mixer based on gravity force and implemented using LCD 3D printing. The 3D-printed mixing part is a slope channel on a disk plane, which creates chaotic advection to improve the mixing performance. We tested gravity mixers with different angled slopes on a centrifugal microfluidic platform, using a high-speed camera to record the mixing process. The gravity mixer achieved sufficient mixing performance in just a few seconds.

## 2. Principle and Design of the Gravity Mixer

**Figure 1:**
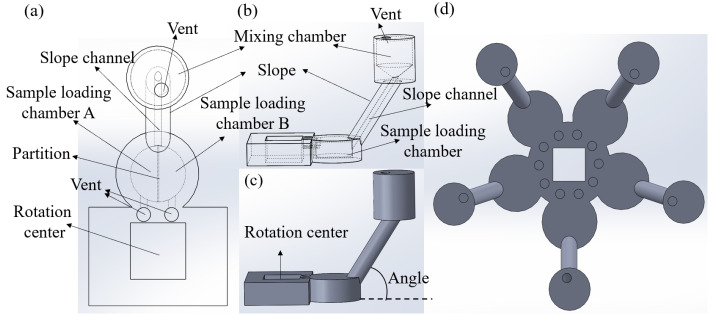
(**a**–**c**) A single gravity mixer: (**a**) the front view of the hidden line visible; (**b**) the right view of the hidden line visible; (**c**) the right view of all areas shaded with edges. (**d**) Schematics of the design with five sets of gravity mixers.

The design of the gravity mixer is illustrated in Figure 1, which was created using Solidworks (Dassault Systems SolidWorks Corp, Waltham, MA, USA). Figure 1a displays the overall layout of the mixer, featuring sample loading chambers A and B, the mixing chamber, the vent, and the slope with an inner channel. The mixing chamber has a funnel structure to generate vortices. The slope channel connecting the sample loading chamber and the mixing chamber can be designed to have different angles. The right view of the gravity mixer is depicted in Figure 1b,c. Initially, sample liquids A and B are loaded into the sample loading chambers separately. The mixer is then installed on the centrifugal platform for testing. When the platform’s rotational speed reaches the burst frequency, the centrifugal forces propel the liquids from loading chambers A and B into the mixing chamber. After the rotation stops, the fluid sample flows back to the loading chambers, creating chaotic advection to enhance mixing. This mixing cycle is repeated multiple times for optimal mixing performance. Figure 1d shows a design with five sets of gravity mixers, allowing for the simultaneous mixing of multiple samples.

**Figure 2:**
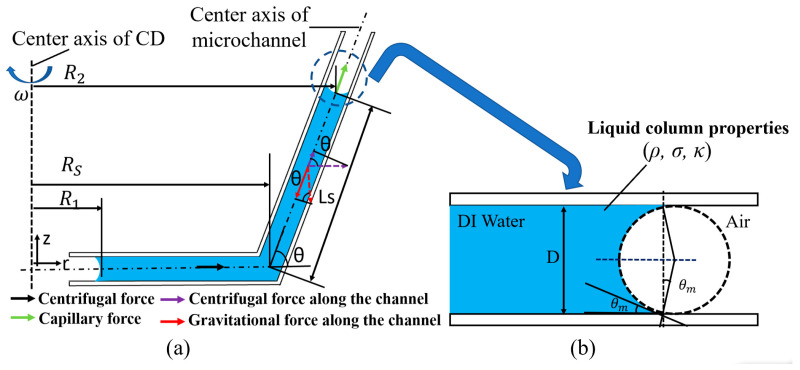
(**a**) A schematic image of the applied force on the liquid column in the slope. (**b**) A schematic image of the liquid meniscus in the connection channel for capillary pressure calculation [44].

Additionally, for an LCD 3D printer, printed layers overlapping on the top of another one can create layer steps. Then, these stacking steps without a cavity can produce roughness for microchannels. Three-dimensionally printed resin microchannels have multiple uniform layers, and the surface of every fine layer does not show a distinct roughness. In this work, the effect of roughness was not studied. Figure 2 graphically demonstrates the forces acting on the liquid column. The movement of the liquid is influenced by several inertial forces, including centrifugal, Coriolis, and Euler forces in the micro-channel, which are caused by the CD’s angular velocity. However, in our design, the magnitude of the centrifugal force acting on the liquid column is much greater than that of the Coriolis and Euler forces, since the liquid column has a negligible linear velocity and angular acceleration. Here, the centrifugal force ($P_{centrifugal}$) on the liquid column can be expressed as follows:

(1) $$P_{centrifugal}=\frac{1}{2}\rho \omega^{2}\left({R_{2}}^{2}-{R_{1}}^{2}\right),$$

and

(2) $$R_{2}=R_{s}+l_{s}cos\theta ,$$

where R_1_ and R_2_ are the length of the liquid column tail and head positions from the CD center axis, ρ is the liquid density, and ω is the angular velocity, respectively. Concurrently, the gravitational force and capillary force can affect the flow of the vertical liquid column in the slope. The gravitational force ($P_{gravitional}$) applied to the liquid column on the slope can be described by Equation (3):

(3) $$P_{gravitional}=\frac{1}{2}\rho gl_{s}sin\theta ,$$

where g is the gravitational acceleration, θ is the angle of slope, and $l_{s}$ is the liquid column length on the slope (shown in Figure 2).

The capillary force ($P_{capillary}$) is calculated using Equation (4),

(4) $$P_{capillarty}=\sigma \kappa ,$$

where σ represents the liquid surface tension and κ represents the measured liquid meniscus curvature. As shown in Figure 2b, the liquid has a concave surface meniscus in the microchannel. The liquid curvature $\left(\kappa =\frac{2cos\theta_{m}}{D}\right)$ is determined by the measured the contact angle ($\theta_{m}$) and microchannel inner diameter (D).

The liquid column will move until the centrifugal, gravitational, and capillary forces are in equilibrium in the microchannel of the slope. Based on this theory, when the liquid column is within a static state along the microchannel of the slope, an equivalent equation can be expressed as follows:

(5) $$P_{centrifugal\;1}+P_{centrifugal\;2}+P_{capillary}=P_{gravitational},$$

where $P_{centrifugal\;1}$ and $P_{centrifugal\;2}$ are the centrifugal forces applied to the liquid column in the microchannel. As shown in Figure 2a, a slope valve section schematic shows the force analysis diagram for the force equilibrium. $P_{centrifugal\;1}$ affects the liquid column from the column head (R_1_) to the slope start point (Rs), and $P_{centrifugal\;2}$ affects the liquid column from Rs to the column tail (R_2_). So, after substituting the formula into Equation (5), the equivalent equation at the slope can be written as follows:

(6) $$\frac{1}{2}\rho \omega^{2}\left[\left({R_{s}}^{2}-{R_{1}}^{2}\right)+cos\theta \left({R_{2}}^{2}-{R_{s}}^{2}\right)\right]+\sigma \kappa =\rho gl_{s}sin\theta .$$

Then, the angular velocity, ω, which is also commonly named the burst frequency, can be defined as follows:

(7) $$\omega \left[RPM\right]=\frac{30\sqrt{2}}{\pi }\sqrt{\frac{gl_{s}sin\theta -\sigma \kappa /\rho }{\left({R_{s}}^{2}-{R_{1}}^{2})+cos\theta ({R_{2}}^{2}-{R_{s}}^{2}\right)}}.$$

Using Equation (7), the burst frequency ω of the gravity mixers can then be estimated.

## 3. Materials and Fabrication

**Figure 3:**
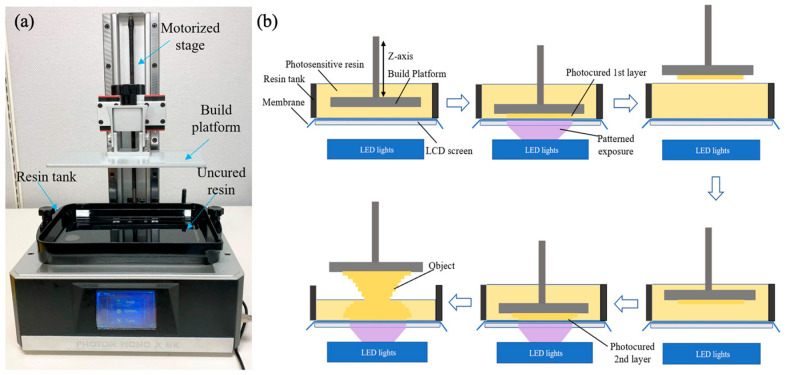
Liquid crystal display (LCD) stereolithographic (SLA) three-dimensional (3D) printing system and the working principle. (**a**) Photograph of the LCD SLA 3D printer. (**b**) Step-by-step workflow of the LCD 3D printer.

To fabricate the 3D-printed microfluidic parts, Solidworks was used to design the parts, which were then converted into STL files and sliced using Photon Workshop. The designed models were converted into a printing code for 3D printing using an ANYCUBIC LCD 3D printer. The horizontal resolution of the ANYCUBIC printer was 34 μm (manufacturer datasheet). Because of its strong stability and good transparency, a 405 nm UV-curable clear resin was used. This UV-cured resin has a good biocompatibility after the material fully undergoing the transition from a liquid status into a solid status [45]. Figure 3a illustrates the schematic layout of the LCD 3D printing system (specifically the ANYCUBIC 6K used in this research). The system comprises several major components: an LCD screen that projects sliced images, a resin tank for loading photosensitive resins, and a motorized build platform that attaches the printed samples. The LCD ANYCUBIC 6K utilizes a bottom-up 3D printing approach, which offers advantages such as shorter curing time and higher vertical resolution compared to the top-down SLA 3D printing method. The step-by-step workflow of the bottom-up LCD 3D printer is shown in Figure 3b. Before initiating the printing process, the STL file, which contains the 3D model of the designed object, is added to the support material and sliced layer by layer using the slicing software (CHITUBOX, Shenzhen, China). Parameters such as layer height, bottom layer count, and exposure time can be adjusted using the slicing software. During the printing process, the build platform moves down via a motorized stage until it is submerged in the photosensitive resins within the resin tank. The distance between the build plate and the membrane at the bottom of the resin tank is defined as the height of the first layer. The sliced image of the first layer is then projected onto the illuminated LCD screen. Only the specific area designated for curing is exposed to UV light emitted by arrays of LEDs. The transparent membrane, made from a non-adhesive Teflon film, allows for easy separation from the cured resin and is attached to the bottom of the build platform. After curing the first layer of the object, the build platform moves up to release the printed layer from the membrane. It then moves down to a specific position where the gap between the bottom of the layer and the membrane defines the height of the second layer. This process (including projection of specific patterns and curing of corresponding layers) is repeated until the 3D-printed gravity mixer is completed.

**Figure 4:**
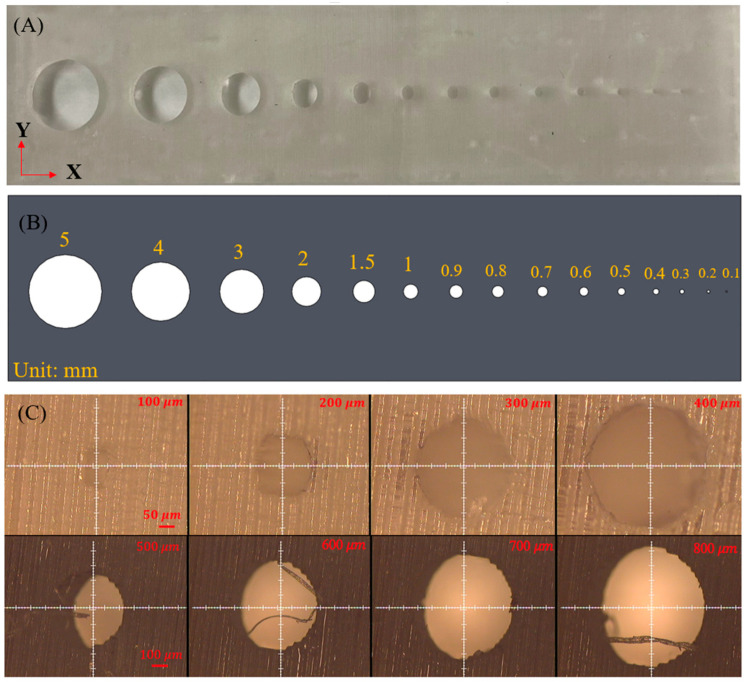
(**A**) Photo image of the 3D-printed micronozzles with different sizes of micro-holes; (**B**) schematic design of 3D micronozzles with different sizes of micro-holes (**C**). Microchannel-designed sizes with diameters of 100 μm, 200 μm, 300 μm, 400 μm, 500 μm, 600 μm, 700 μm, and 800 μm, respectively.

**Figure 5:**
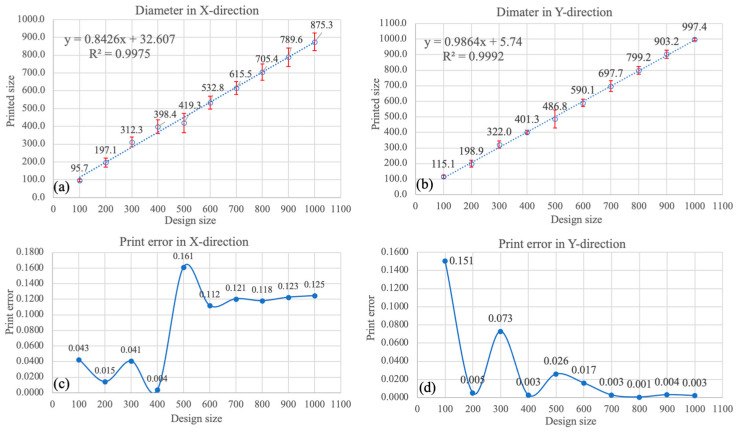
(**a**,**b**) Relation between the fabricated size and designed size of the microchannels. (**c**,**d**) Print error between the fabricated size and designed size of the microchannels.

Figure 4A displays the schematic design of microchannels with different sizes. A total of five circular microchannel designs were batch-printed. The corresponding microscope images of microchannels ranging from 100 μm to 800 μm are depicted in Figure 4C. For the size of microchannels ranging from 100 μm to 400 μm, the 10× lens was employed. For the size of microchannels ranging from 400 μm to 800 μm, the 5× lens was employed. The overall effect, accuracy, and resolution of the printed objects appeared excellent. To evaluate the accuracy of the printed microchannels, the diameters of the printed microchannels were measured for the five printed objects with the same microchannel design. The average diameter values of the microchannels in the X and Y directions are shown in Figure 5a,b, respectively. The corresponding print errors are illustrated in Figure 5c,d. The results show that the fabricated size has a fluctuating and larger print error in the X and Y direction under the size of 700 μm. In contrast, the accuracy in both directions tends to be stable above 700 um, with a print error less than 0.4% in the Y direction. Considering our specific design of the mixer, in which the smallest microchannel size is larger than 700 μm, the print error is expected to be around 12% in the X direction and less than 0.4% in the Y direction. These results demonstrate that the LCD 3D printer exhibits excellent accuracy in the Y direction. Therefore, the print error in the Y direction was not considered in our study. It is worth noting that since we used transparent clear resin, which is susceptible to overexposure, the experimental results may vary with different settings such as layer thickness, exposure time, and the addition of support material.

**Figure 6:**
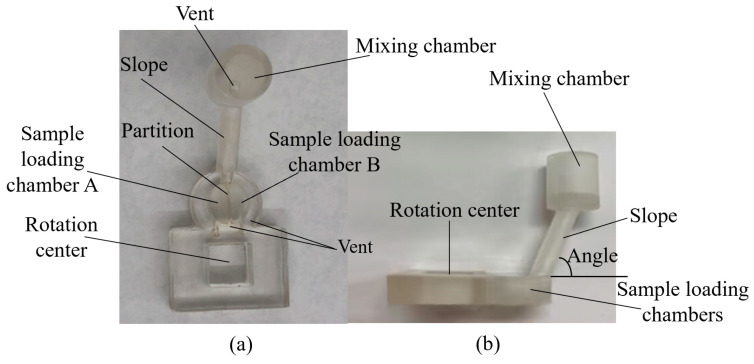
The photo image of a 3D-printed gravity mixer: (**a**) the top view; (**b**) the side view, angle = 60°.

The 3D-printed gravity mixer with a slope angle of 60° is shown in Figure 6. The fabrication of each prototype device took about 1 h and 50 min regardless of the different slope angles. Gravity mixers with different slope angles (20°, 30°, 40°, 50°, 60°, 70°, 80°) were fabricated for the experiments. Several prototypes can be designed in each SolidWorks model and fabricated in a single printing batch. After printing, the mixers were washed in isopropyl alcohol (IPA) for 15 min and the support material was removed using professional scissors. The mixers were then air-dried for half an hour and cured for 3 min using a UV cure station.

## 4. Experimental Results and Discussions

**Figure 7:**
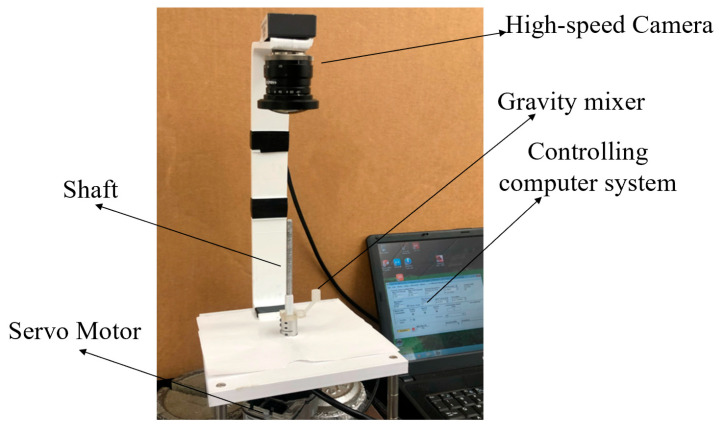
The experimental setup of the centrifugal platform with the 3D microfluidic cartridge mounted.

Figure 7 shows the centrifugal platform setup we used in the lab to test the gravity mixers. The microfluidic mixer was attached to a shaft powered by a DC servo motor, which was controlled using a laptop computer. A high-speed camera with a frame rate of 80 fps (Pixelink, Rochester, NY, USA) was installed above the centrifugal system to monitor the mixing process in real time. Various experiments were conducted to analyze the mixing performance of the gravity mixer at different slope angles.

**Figure 8:**
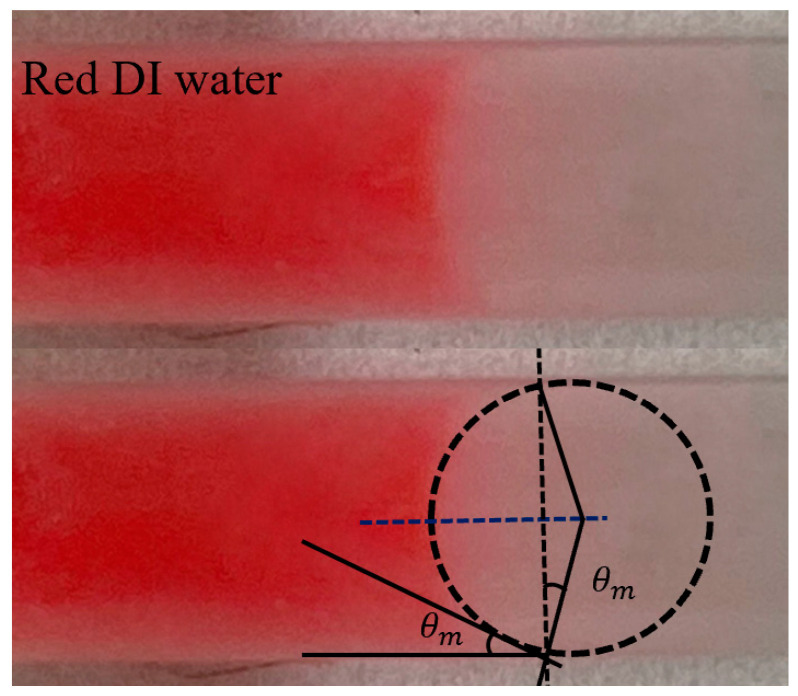
Optical image of liquid meniscus in the connection channel for capillary pressure calculation.

**Figure 9:**
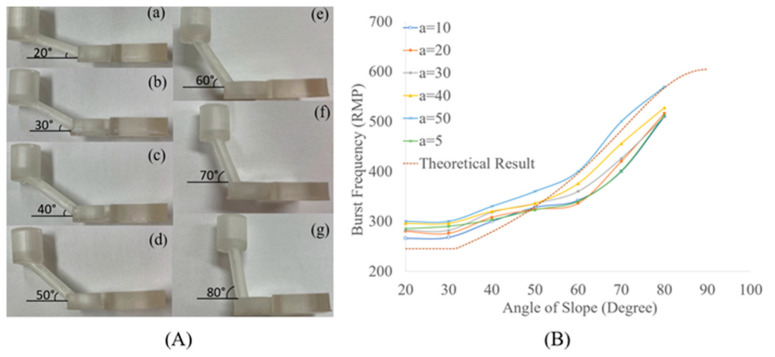
(**A**) Three-dimensionally printed SLA gravity mixers with different angles; (**B**) burst frequency (the minimal rotational frequency when the liquid column head starts to flow into the mixing chamber) versus the slope angle variation.

The image in Figure 8 was captured using an iPhone with a 100× pocket microscope camera lens (APEXEL, Littleton, CO, USA). With a diameter (D) of 2 mm, the capillary force can be calculated using Equation (4). According to Equation (6), the critical angle is calculated to be 33.02° when the component of gravity force equal to capillary force ($gl_{s}sin\theta -\frac{\sigma \kappa }{\rho }=0$). In this design, the capillary force dominates in the slope for angles less than 33°. We conducted experiments to test the burst frequencies at different slope angles of the gravity mixer. The 3D-printed gravity mixers with different angles are shown in Figure 9A. As shown in Figure 9B, the brown dashed-dotted line represents the theoretical burst frequency for different slope angles, and the other six colored lines represent experimental results with angular acceleration variations (5, 10, 20, 30, 40, and 50 rpm/s). When the slope angle is less than 33.02°, the capillary force dominates in the slope channel, and the burst frequency (angular velocities) will not exceed 245 rpm, which is consistent with Figure 9B. This is because the burst frequency can be assumed to be an imaginary number for the given input when the component of gravity force is less than capillary force ($gl_{s}sin\theta -\frac{\sigma \kappa }{\rho }<0$) [44]. In this situation, the adhesive force between the liquid interface and loading chamber inner surface, and the capillary forces due to liquid–solid interactions mainly affect the burst frequency. When $gl_{s}sin\theta -\frac{\sigma \kappa }{\rho }>0$, regardless of angular acceleration variations (i.e., 5, 10, 20, 30, 40, and 50 rpm/s), the burst frequency increases with an increasing slope angle, changing from 280 to 600 rpm for slope angles increasing from 30° to 80°. The experimental results shown in Figure 9B are consistent with the theoretical analysis.

**Figure 10:**
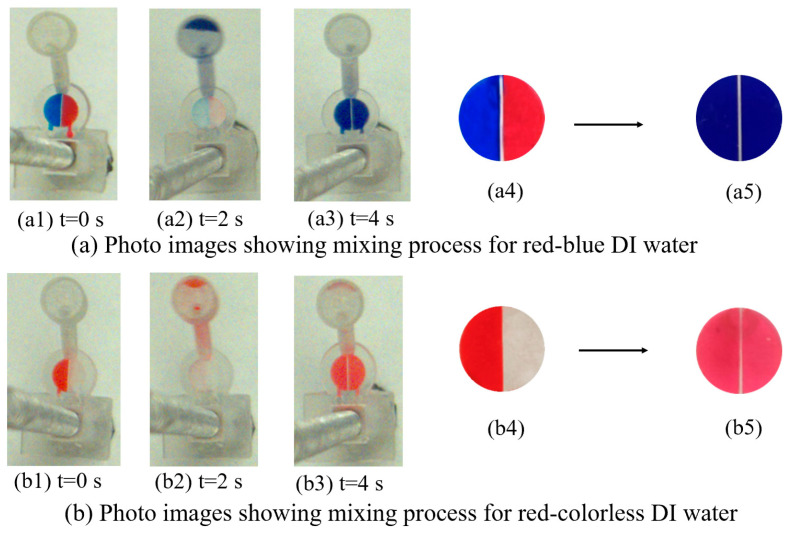
Photo images (**a**) showing the mixing process for red–blue DI water. Photo images (**b**) showing the mixing process for the red–colorless DI water. The photos in (a4,a5) and (b4,b5) were taken with an iPhone to obtain a better resolution for accurate analysis in the next steps.

The entire mixing process for red–blue-dyed deionized (DI) water and red–colorless DI water is displayed in Figure 10a,b. At the beginning of the mixing experiments, 100 µL of red and blue DI waters were separately injected into loading chambers A and B. Experiments were conducted using dyed DI water to facilitate observation under a high-speed camera. The gravity mixer was designed in such a way that the loading chamber was located closer to the shaft, while the mixing chamber was toward the edge of the platform along the radial direction. The gravity mixer was rotated in a counterclockwise direction. For the tested gravity mixer, the slope angle was 60° and the burst frequency was 400 RPM. When the rotational speed of the gravity mixer was increased to 400 RPM, the red and blue DI waters in loading chambers A and B were released, flowed through the slope channel, and entered the mixing chamber, as shown by the sequential photo images in Figure 10(a1,a2).

When the mixer stopped rotating, the mixing DI water flowed back to the loading chambers via the slope channel due to gravity, as shown in Figure 10(a3). The entire mixing process took only 4 s for the gravity mixer with a 60° slope angle, and was recorded by a high-speed camera at a rate of one picture per second. The photos in Figure 10(a1–a4) show the color change of DI water in loading chambers A (blue) and B (red) before mixing (Figure 10(a4)), which became purple (Figure 10(a5)) for both loading chambers after mixing, indicating good mixing performance.

To further analyze the mixing performance, the same experiment steps were conducted using colorless and red-dyed DI water, and the results are displayed in Figure 10(b1–b3). Figure 10(b4,b5) shows the change in color in loading chamber A (red) and loading chamber B (colorless) before mixing (Figure 10(b4)), whereby it became red (Figure 10(b5)) for the both loading chambers after mixing.

From Figure 10, it is observed that the two liquids mixed effectively. Quantitative analysis is necessary to determine the mixing performance of the gravity mixer. Standard deviation (σ) is a widely used method for analyzing mixing performance. The grayscale distribution of the colorless and red-dyed DI water is approximately from 200–255 and 0–50, respectively. To assess the effectiveness of the gravity mixer, the standard deviation of the pixel intensity in images of the mixed liquids in the loading chambers for red–colorless DI water mixing was calculated to numerically represent the mixing effectiveness. The standard deviation (σ), which measures the dispersion of data values, can be used to evaluate mixing performance. A low σ value in the histogram indicates better mixing performance, with data points clustered closely around the mean value. Conversely, a high σ value indicates poorer mixing performance, with data points widely distributed. The software programs “Image J” (NIH Image, Bethesda, MD, USA) and “Origin 8.5” (OriginLab, Northampton, MA, USA) were used to process and analyze images of the final mixed liquid in the loading chamber, obtaining the σ and distribution of the gray intensity histogram. For the final mixed liquids shown in Figure 10(b5), the region of interest was the loading chamber, which was digitally cut out for analysis. The gray intensities of all the pixels were obtained, and the σ of each image was calculated for gravity mixers with slope angles of 40°, 50°, 60°, 70°, and 80°. Figure 8 presents the results of the σ and histogram for the final mixed liquid in the loading chamber.

**Figure 11:**
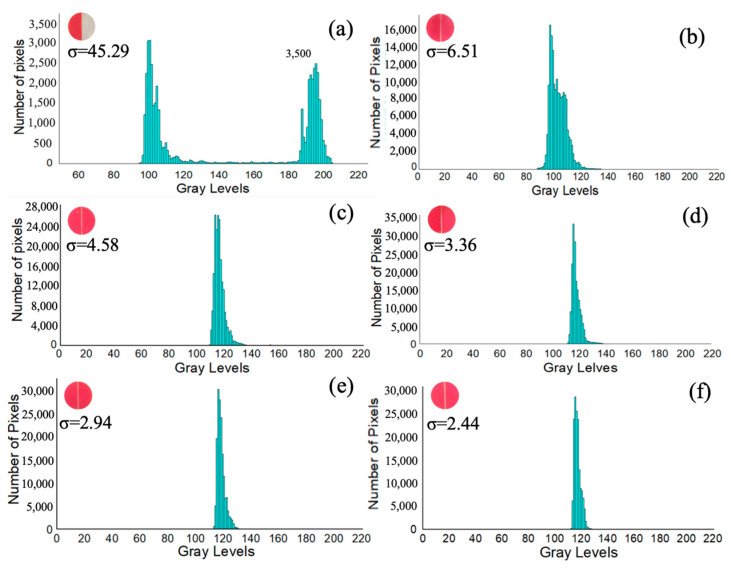
Histogram and σ of the area of interest (**a**) before mixing; (**b**) mixer with a 40° slope angle after mixing; (**c**) mixer with a 50° slope angle after mixing; (**d**) mixer with a 60° slope angle after mixing; (**e**) mixer with a 70° slope angle after mixing; (**f**) mixer with an 80° slope angle after one mixing cycle.

The x-axis of the histogram represents the gray levels and the y-axis represents the number of pixels in that specific tone. Figure 11a shows the distribution of pixels for the red and colorless DI water in the loading chambers before mixing for the gravity mixer, with gray levels from 100 to 130 and 170 to 207, respectively. However, these gray levels do not fall into the expected ranges of 0–50 and 200–255 due to the clear resin having its own color after curing. The distribution of the gray levels was more dispersed and less concentrated before mixing in contrast to the gravity mixers with different slope angles after mixing, as shown in Figure 11b–f. The histogram distributions of different slope angles with 40°, 50°, 60°, 70°, and 80° are from 87 to 138, from 108 to 134, from 112 to 138, from 113 to 135, and from 112 to 132, respectively.

As can be observed from the experimental data, the histogram distributions became less dispersed and more concentrated with an increasing slope angle, indicating improved mixing efficiency of the gravity mixer. The initial σ value before mixing was 45.29, which decreased after mixing for the gravity mixers (6.51 for 40°, 4.58 for 50°, 3.36 for 60°, 2.94 for 70°, 2.44 for 80°) with an increasing slope angle.

**Figure 12:**
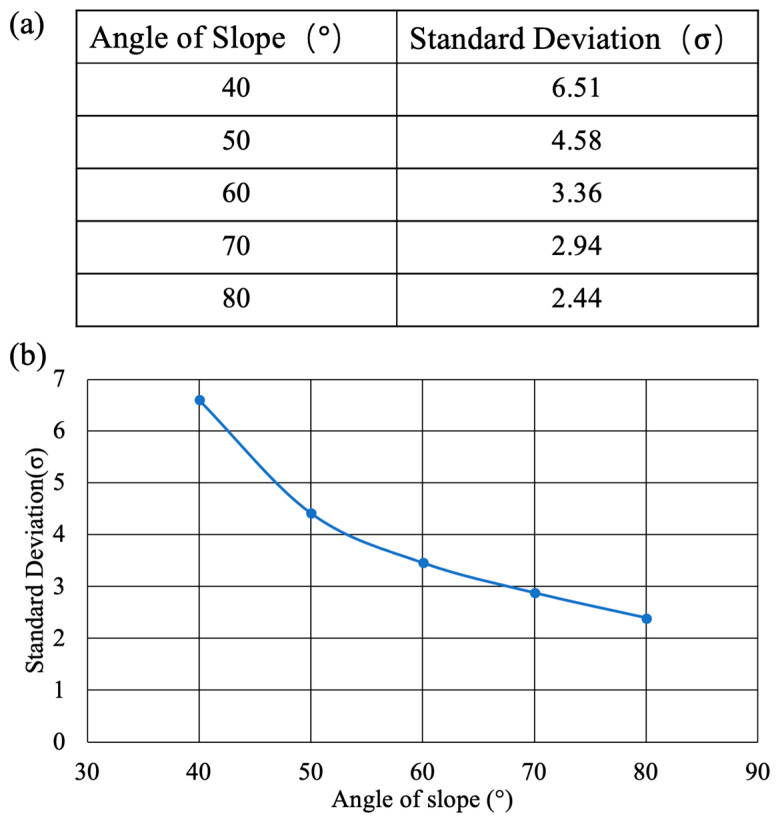
(**a**,**b**) The change in standard deviation with an increase in the angle of the slope.

**Figure 13:**
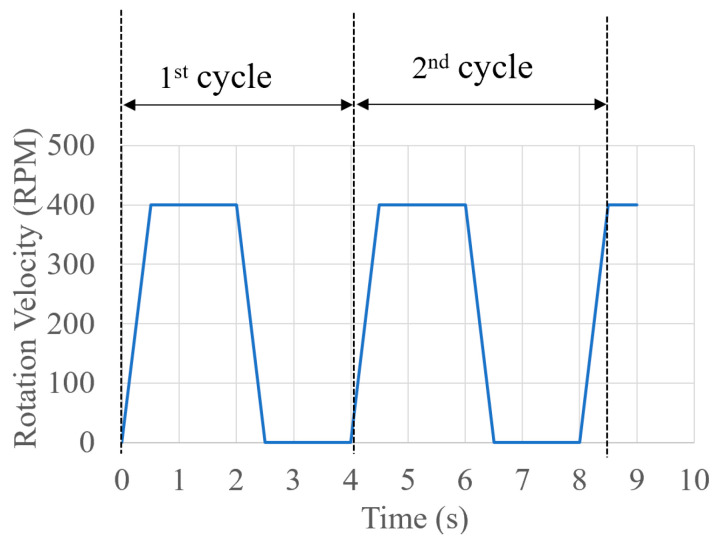
The rate of change in rotational velocity of the disc during the mixing cycle for a gravity mixer with 60°.

Additionally, gravity mixers with different sizes were fabricated, with five pieces for each size, and experiments were repeated five times for each gravity mixer size. The corresponding results of average mixing efficiency for each gravity mixer size are shown in Figure 12a,b. The average σ of the gravity mixers with different slope angles was 6.61 (40°), 4.42 (50°), 3.46 (60°), 2.88 (70°), and 2.39 (80°), respectively. A smaller σ indicates better mixing efficiency. Therefore, the mixing efficiency of the gravity mixer with different slope angles is different, becoming higher with an increasing slope angle due to the burst frequency growing larger, resulting in quick chaotic advection for faster mixing. In addition, the mixing process of the gravity mixer is highly repeatable due to the reciprocating movement of the liquids under the influence of gravity, centrifugal force, and capillary force. Figure 13 displays the corresponding mixing cycle times and rotational velocities for the gravity mixer with a 60° slope angle. The entire mixing experiment was repeated five times for each mixing cycle for the gravity mixer.

**Figure 14:**
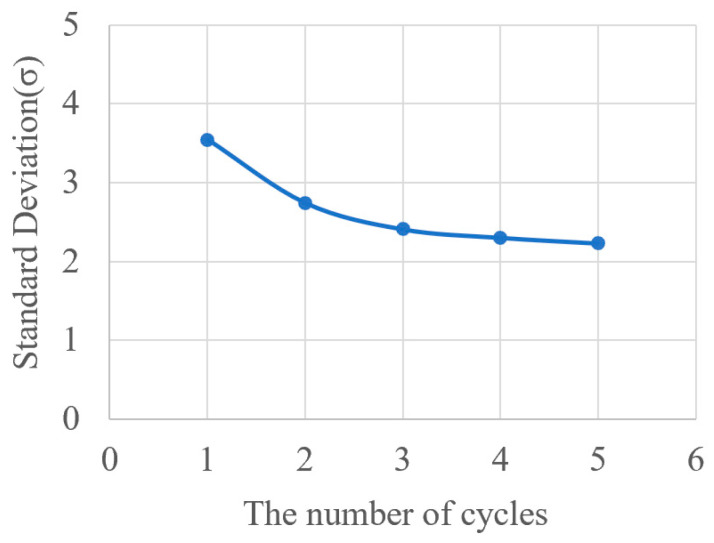
The change in standard deviation with the times of the repeated mixing process for a mixer with 60°.

The variation in σ as a function of the number of mixing cycles is shown in Figure 14. The results indicate that the standard deviation σ initially decreased rapidly as mixing cycles increased, then became steady as the number of cycles further increased. The experimental results demonstrate that an efficient mixing result with σ = 2.41 can be achieved after three cycles (12 s) in the gravity mixer. After that, further increasing in the mixing cycles produced only slight improvement in mixing efficiency. In comparison, the jet mixer has a mixing time of 5 s and σ of 3.56 [41], the Y-shaped mixer has a mixing time of 5 s with σ of 13.77 [41], and the moment of inertia mixer has a mixing time of 18 s with σ of 3.04 [46]. Overall, the gravity mixer can obtain better mixing results in a shorter time.

It is important to acknowledge that the gravity mixer presented in this study is only effective for slope channels with a diameter in the millimeter range. The flow of the liquid in the sloped channel is influenced by its diameter (D), with a decrease in the diameter resulting in an increase in the capillary pressure value, $P_{capillary}\left(\sigma \left(\frac{2cos\theta_{m}}{D}\right)\right)$.

**Figure 15:**
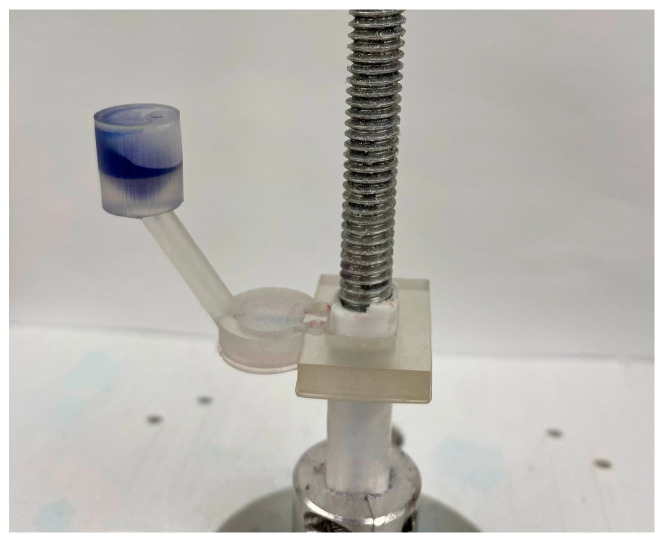
The gravity mixer with a 700 μm diameter slope channel after mixing.

The critical diameter (778 μm at θ = 60°), as obtained using the equation ($gl_{s}sin\theta -\frac{\sigma \kappa }{\rho }=0$), plays a crucial role. If the diameter of the slope channel is less than 778 μm for a slope angle of 60°, the sample liquids in the mixing chamber cannot flow back into the loading chamber due to the surface tension force at the liquid–air interface, as depicted in Figure 15. Additionally, in this study, we mainly focus on water–water mixing, such as DI water (surface tension (2 mN/m) and viscosity (0.89 mPa·s). The material used for fabricating the gravity mixer is known to be stable in saline water, and therefore has no biocompatible issues for any applications involving saline water. It is not suitable for liquids with alcohol content. Noteworthily, the use of image analysis to estimate mixing performance is subject to limitations such as camera resolution, the unsmooth inside surface of the loading chamber resulting from the limitations of the 3D printer, and the irregular shape of the liquid samples in the loading chamber. Nevertheless, the σ of the gray intensities provides a useful estimation for mixing performance. Here, the design and fabrication of the gravity mixer and its mixing performance have been demonstrated. Additionally, clear UV resin was selected as the building material for visualization purposes only. Many other materials such as tough resin, water wash resin, plant-based rapid resin, and ABS-like resin, etc., in ANYCUBIC LCD and even more types of materials and corresponding 3D printing technologies can be selected based on specific applications and needs. One potential application of the gravity mixer is in competitive enzyme-linked immunosorbent assays (ELISAs). The gravity mixer may also be used in chemical reactions, and biological and medical applications.

## 5. Conclusions

In this study, a gravity mixer based on inertial and gravity forces was designed, fabricated using LCD 3D printing technology, and tested. The mixing efficiency of the gravity mixer was evaluated using digital analysis of photo images of the fluid sample. The mixing efficiencies of the gravity mixers with different slope angles were studied, with the gravity mixer with a 60° slope angle achieving an average σ of 3.46 for colorless DI water and red dye-colored DI water in less than 4 s. The mixing process was repeatable, with the gravity mixer reaching an ideal mixing effect with a standard deviation value of σ at 2.41 after three cycles. The study showed that this chaotic mixing method effectively mixed liquids. This new mixing technology can be easily integrated in centrifugal fluidic platforms for a wide variety of applications. The study also demonstrated that 3D printing technology offers opportunities for future applications and the integration of complex Lab-on-CD structures.

## Data Availability

The data presented in this study are available on request from the corresponding author (accurately indicate status).
